# *KRAS* Loss of Heterozygosity Promotes MAPK-Dependent Pancreatic Ductal Adenocarcinoma Initiation and Induces Therapeutic Sensitivity to MEK Inhibition

**DOI:** 10.1158/0008-5472.CAN-23-2709

**Published:** 2024-10-16

**Authors:** Sigrid K. Fey, Arafath K. Najumudeen, Dale M. Watt, Laura M. Millett, Catriona A. Ford, Kathryn Gilroy, Rosalin J. Simpson, Kathy McLay, Rosanna Upstill-Goddard, David Chang, William Clark, Colin Nixon, Joanna L. Birch, Simon T. Barry, Jennifer P. Morton, Andrew D. Campbell, Owen J. Sansom

**Affiliations:** 1 Cancer Research UK Scotland Institute, Glasgow, United Kingdom.; 2 School of Cancer Sciences, University of Glasgow, Glasgow, United Kingdom.; 3 Wolfson Wohl Cancer Research Centre, Glasgow, United Kingdom.; 4 Bioscience, Oncology R&D, AstraZeneca, Cambridge, United Kingdom.

## Abstract

Pancreatic cancer is characterized by the prevalence of oncogenic mutations in *KRAS*. Previous studies have reported that altered *KRAS* gene dosage drives progression and metastasis in pancreatic cancer. Whereas the role of oncogenic *KRAS* mutations is well characterized, the relevance of the partnering wild-type (WT) *KRAS* allele in pancreatic cancer is less well understood and controversial. Using *in vivo* mouse modeling of pancreatic cancer, we demonstrated that WT KRAS restrains the oncogenic impact of mutant KRAS and dramatically impacts both KRAS-mediated tumorigenesis and therapeutic response. Mechanistically, deletion of WT *Kras* increased oncogenic KRAS signaling through the downstream MAPK effector pathway, driving pancreatic intraepithelial neoplasia initiation. In addition, in the KPC mouse model, a more aggressive model of pancreatic cancer, lack of WT KRAS led to accelerated initiation but delayed tumor progression. These tumors had altered stroma and an enrichment of immunogenic gene signatures. Importantly, loss of WT *Kras* sensitized *Kras* mutant tumors to MEK1/2 inhibition though tumors eventually became resistant and then rapidly progressed. This study demonstrates the repressive role of WT KRAS during pancreatic tumorigenesis and highlights the critical impact of the presence of WT KRAS in both tumor progression and therapeutic response in pancreatic cancer.

**Significance:**

*KRAS* allelic status impacts pancreatic cancer progression and has the potential to guide effective treatment in a substantial subset of patients.

## Introduction

Oncogenic *KRAS* mutations are common in cancer and almost ubiquitous in pancreatic cancer, with mutations detected in around 95% of patients. This significant prevalence of *KRAS* mutations was historically compounded by a lack of success in direct therapeutic targeting of oncogenic KRAS. However, there has been a recent revolution in this area, with the development and subsequent clinical use, albeit in different indications, of covalent binding inhibitors of KRAS-G12C, such as AMG-510 (sotorasib; ref. 1). Many years of research have furthered our understanding of the cellular and biochemical impact of oncogenic *KRAS* mutations in cancer. This is now being expanded by growing evidence that in addition to oncogenic mutations, allelic balance at oncogenic loci, such as *KRAS*, and the resulting oncogene dosage are critical modifiers of oncogenic signaling. This is not restricted to pancreatic cancer, with similar oncogene allele imbalances detected in cancers of different tissues of origin, such as lung, colorectal, melanoma, breast, and ovarian cancers (2–7). Moreover, copy-number variants are also detected with different driver genes (*NRAS*, *BRAF*, *EGFR*, and *MYC*; refs. 2, 3, 6–9).

In the case of *KRAS*, there is now clear evidence that alterations in allele frequencies or gene dosage at oncogene loci can have a dramatic impact upon tumorigenesis and tumor-associated phenotypes across tumor types (5, 6, 10). *KRAS* allelic imbalances are frequently observed (55%) in mutant *KRAS*–driven cancer across many different tumor types (4). The effect of *KRAS* allelic imbalance upon therapeutic efficacy was observed in a study by Burgess and colleagues (11), in which it was demonstrated that the relative balance between mutant and wild-type (WT) *KRAS* alleles determined clonal fitness and sensitivity to MAPK-targeting therapeutic approaches in acute myeloid leukemias. Allelic imbalances at the *KRAS* locus have previously been reported in pancreatic ductal adenocarcinoma (PDAC), the most common type of pancreatic cancer, and were associated with early tumor progression (5). There is evidence that *KRAS* gene dosage has impact on PDAC evolution, indicated by variations in cell morphology, histopathology, and clinical outcome in patients. Moreover, copy-number alteration of *KRAS*, such as copy-number gain of oncogenic *KRAS*, loss of heterozygosity (LOH), or allelic imbalance can be beneficial for tumor growth. In pancreatic cancer, increased *KRAS* gene dosage is associated with early tumorigenesis, tumor progression, and increased incidence of metastasis *in vivo* (5).

There is growing evidence that *KRAS* allelic imbalance might significantly affect mutant KRAS signaling through increased oncogenic *KRAS* gene dosage relative to the WT allele (5, 12). This suggests that there are three distinct types of human *KRAS* mutant cancers, tumors that contain the WT *KRAS* allele and are heterozygous for *KRAS*, tumors in which the mutant *KRAS* allele is exclusively expressed (homozygous), and tumors that have mutant *KRAS* copy-number gain (12). This could conceivably impact tumor initiation and progression and may even modify responses to therapy through enabling intrinsic and acquired resistance.

We demonstrate the functional relevance of WT KRAS and present mechanisms that govern the role of WT KRAS in the processes of tumor initiation and progression in pancreatic cancer. We use genetically engineered mouse models (GEMM) of pancreatic cancer, allowing deletion of WT *Kras*, in combination with preclinical trialing of clinically relevant target therapeutic approaches to carefully dissect each of these features. We demonstrate that the presence of WT KRAS restrains mutant KRAS signaling and that WT KRAS loss results in increased pancreatic intraepithelial neoplasia (PanIN) *in vivo*. Importantly, we show that loss of WT *Kras* alters the progression of pancreatic tumors and leads to increased immune cell infiltration in preclinical models of pancreatic cancer. Using late-stage treatment intervention studies, we show that loss of WT *Kras* enhances sensitivity to MEK1/2 inhibition *in vivo*. This study highlights the impact of *Kras* allelic imbalance and the critical role of WT KRAS in initiation, progression, and therapeutic response in pancreatic cancer.

## Materials and Methods

### International Cancer Genome Consortium/Australian Pancreatic Cancer Genome Initiative RNA sequencing patient dataset

Transcriptomic profiling and molecular characterization of the International Cancer Genome Consortium (ICGC), Pancreatic Cancer - Australian Collection (PACA-AU) was performed as previously described (13). Copy-number calls for samples included in the Pan-Cancer Analysis of Whole Genomes (PCAWG) project were obtained from the International Cancer Genome Consortium. Samples were defined as either *KRAS* balanced or *KRAS* imbalanced. Samples classed as *KRAS* balanced had an equal number of copies of the major allele and minor alleles, whereas samples classed as *KRAS* imbalanced had more copies of the major allele compared with the minor allele.

### Mouse models and experiments

Experiments using GEMMs were performed according to the UK Home Office regulations (licenses 70/8646 and PP3908577) under approval by the Animal Welfare and Ethical Review Board of the University of Glasgow. The *in vivo* studies were performed using the KPC mouse model (14). The alleles were used as follows—*Pdx1*-Cre (15)*, Kras*^LSL-G12D^ (16), lox-stop-lox-*Trp53*^R172H^ (17), and *Kras*^fl^ (18). The smallest sample size that could give a significant difference was chosen (specified in relevant figure legends), in accordance with principles of reduction, refinement, and replacement of animals in science (3Rs). No formal randomization was used, and the experimenter was blinded to genotypes. Mice were bred on a mixed genetic background, in which control and experimental animal colonies were of the same genetic background, confirmed by genotyping array (MiniMUGA, Transnetyx). Mice of both sexes were used for tumor studies and monitored at least three times weekly and sampled when exhibiting clinical signs of PDAC (abdominal swelling, jaundice, hunching, piloerection, or loss of body conditioning).

### Tumor monitoring

High-resolution ultrasound imaging was performed using a Vevo 3100 system [with MX550D (25–55 mHz)] transducer (VisualSonics). To identify tumor-bearing animals, mice were assessed twice weekly for palpable abdominal masses, and if abdominal mass was present, pancreatic tumor burden was confirmed. Tumor size at enrollment was measured by high-resolution ultrasound imaging. Tumor growth was then monitored once weekly by ultrasound imaging until reaching clinical endpoint using VeVo LAB (Fujifilm, Visual Sonics, VeVo LAB 1.7.1).

### *In vivo* treatment experiments

For drug treatment studies, pancreatic malignancy was confirmed by abdominal palpation and ultrasound imaging. Mice were randomly assigned to cohorts. Mice were treated from palpable pancreatic tumor burden assessment until reaching clinical endpoint or from indicated timepoints (as indicated in the figure legends). The MEK1/2 inhibitor (AZD6244/selumetinib) was administered with a dose of 25 mg/kg^-1^ twice daily via oral gavage in a vehicle of 0.5% hydroxypropyl methylcellulose (HPMC) and 0.1% Tween-80.

### PanIN scoring

For PanIN scoring, mice were sampled at indicated time points, and PanINs were scored from whole hematoxylin and eosin sections and normalized to mm^2^ pancreas of the whole section. PanINs were scored according to the classification of PanINs by Johns Hopkins University’s Department of Pathology (19, 20).

### Immunohistochemistry

Immunohistochemistry was performed on formalin-fixed pancreatic sections using standard protocols. Hematoxylin and eosin staining was performed using standard protocols. Picrosirius red staining technique was used to stain collagen within tissue sections, as described previously (19). Primary antibodies used for IHC were as follows: phospho-ERK1/2 (1:400, Cell Signaling Technology, #9101), cMYC (1:200 Abcam, #ab32072), p53 (1:100, Cell Signaling Technology, #2524), α-smooth muscle actin (αSMA; 1:25,000, Sigma-Aldrich, #A2547), p21 (1:150, Abcam, #107099), Ki67 (1:100, Thermo Fisher Scientific, #RM-9106), Tenascin C (1:300, Sigma-Aldrich, #T3413), DUSP6 (1:250, Abcam, #Ab76310), CD3 (1:100, Abcam, #Ab16669), CD4 (1:500, eBioscience, #14-9766-82), and CD8 (1:500, eBioscience, #14-0808-82). *In situ* hybridization (ISH; RNAscope) was performed according to the manufacturer’s protocol (Advanced Cell Diagnostics, RNAscope 2.0 High Definition–Brown) for *Dusp5* (Bio-Techne, #475698), *Dusp6* (Bio-Techne, #429321), and *cmyc* (Bio-Techne, #508808). Sections were counterstained with hematoxylin and covered by a coverslip using distyrene, plasticizer, and xylene (DPX) mountant (CellPath).

### Assaying proliferation *in vivo*

Proliferation levels were assessed by measuring 5-bromo-2'-deoxyuridine (BrdU) incorporation. Mice were injected intraperitoneally with 250 μL of BrdU (Amersham Biosciences) 2 hours before being sacrificed. IHC staining for BrdU was then performed using an anti-BrdU antibody (1:200, BD Biosciences, #347580).

### Assaying subconfluent migration *in vitro*

For subconfluent migration assay, 2 × 10^4^ cells were seeded per well in 6-well plates, and images were acquired in phase contrast (Nikon Ti-E) every 20 minutes for 16 hours at ×10 magnification (10 × /0.30 Plan Fluor Ph1, Nikon). Cell speed was calculated using single-cell tracking via Manual Tracking plugin in ImageJ (21).

### Western blotting

Snap-frozen PDAC tissue was homogenized in RIPA lysis buffer (Tris pH 7.4, NaCl, Triton X-100, SDS, and dH_2_O) using a Precellys homogenizer. Lysates were centrifuged for 10 minutes at full speed. Protein concentration in the supernatant was determined using BCA Protein Assay Kit (Thermo Fisher Scientific, #23225). Samples were boiled in loading buffer for 5 minutes at 95°C and allowed to cool down to room temperature prior to loading, and 20 μg of protein were separated on 4% to 12% gradient precast gel (Novex, #NP0322PK2) and run in 3-(N-morpholino)propanesulfonic acid (MOPS) running buffer. Samples were transferred onto polyvinylidene difluoride membranes (Millipore, #IPFL00010). The membranes were blocked in 5% BSA (Sigma-Aldrich, #A9647) in TBS with Tween (TBST) for 1 hour at room temperature. Primary antibodies were incubated in 5% BSA in TBST or 5% milk in TBST at 4°C overnight. The primary antibodies were used as follows: KRASG12D 1:1,000, Cell Signaling Technology, #14429; β-actin 1:2,000, Sigma-Aldrich, #A2228; ERK1/2 1:1,000, Cell Signaling Technology, #4695; pERK1/2 1:1,000, Cell Signaling Technology, #9101; MEK1/2 1:1,000, Cell Signaling Technology, #8727; pMEK1/2 1:1,000, Cell Signaling Technology, #2338; AKT 1:1,000, Cell Signaling Technology, #9272; pAKT (Ser473) 1:2,000, Cell Signaling Technology, #4060; PTEN 1:1,000, Cell Signaling Technology, #9559; and DUSP6 1:1,000, Abcam, #ab76310. The Secondary antibody (α-rabbit Dako, #P0448; α-mouse Dako, #P0447) was diluted 1:2,000 in 5% BSA/TBST and incubated for 1 hour at room temperature. Membranes were incubated with enhanced chemiluminescence plus working reagent (Thermo Fisher Scientific, catalog no. 32132) for 2 minutes and bands were detected using a ChemiDoc imager (Bio-Rad). Membranes were briefly washed with TBST and stripped with Replot Plus (Millipore, #2504) for 10 minutes at room temperature and rinsed with TBST.

### RAF–RAS-binding domain assay (RAS pulldown activation assay)

Snap-frozen PDAC tissue was homogenized in lysis buffer using a Precellys homogenizer. Lysates were centrifuged for 10 minutes at full speed at 4°C and transferred to a new tube. Protein concentration in the supernatant was determined using BCA Protein Assay Kit (Thermo Fisher Scientific, #23225), and 500 μg of protein were used for the RAS-binding domain (RBD) assay. The following steps were performed using Cytoskeleton RAS Activation Assay Biochem Kit (Cytoskeleton, # BK008) according to the manufacturer’s instructions. Briefly, 30 μL of RAF-RBD beads were added to each tube and incubated for 1 hour on a rotator at 4°C. RAF-RBD beads were centrifuged for 1 minute at 5,000 *g* and 4°C. The supernatant was removed, and the beads were washed with 500 μL wash buffer and centrifuged for 3 minutes at 5,000 *g* and 4°C. The supernatant was carefully removed, and 20 μL of 2× Laemmli sample buffer was added to each tube. The samples were boiled to 5 minutes at 95°C. The samples were loaded onto 4% to 12% gradient precast gel (Novex) and processed as Western blotting samples, as described above. GDP-incubated samples served as a negative control, GTPγS served as a positive control, and His-tagged RAS protein served as the control protein. The total protein input served as the loading control.

### mRNA isolation, qRT-PCR, and RNA sequencing

PDAC tissue samples preserved in RNAlater (Sigma-Aldrich, #R0901) were homogenized using a Precellys homogenizer, and RNA was extracted from tumor tissue using QIAGEN RNeasy Kit (Qiagen, #74104) according to the manufacturer’s instructions, including DNase digestion. A measure of 0.5 μg of total RNA was reverse-transcribed using High-Capacity cDNA Reverse Transcription Kit (Thermo Fisher Scientific, #4368814) according to the manufacturer’s instructions. qRT-PCR was performed in technical replicates using QuantiFast SYBR Green RT-PCR Kit (Qiagen, #204156) according to the manufacturer’s instructions. The reaction mixture without a template was run in duplicate as a control. *Gapdh* was used to normalize for differences in RNA input. For RNA sequencing, RNA integrity was analyzed using a NanoChip kit (Agilent RNA 6000 Nano Kit, 5067-1511), and 1 μg of RNA was prepared in water to a final volume of 50 μL.

As previously described, RNA sequencing was performed by the Molecular Technologies Service of the CRUK Scotland Institute using an Illumina TruSeq RNA sample preparation kit and then run on an Illumina NextSeq high-output 75-cycle kit (2 × 36 cycles, paired-end reads, single index). The raw sequence quality was assessed using the FastQC algorithm version 0.11.8. Sequences were trimmed to remove adaptor sequences and low-quality base calls, defined by a Phred score of <20, using the Trim Galore tool version 0.6.4. The trimmed sequences were aligned to the mouse genome build GRCm38.98 using HISAT2 version 2.1.0, and then raw counts per gene were determined using FeatureCounts version 1.6.4. Differential expression analysis was performed using the R package DESeq2 version 1.22.2, and principal component analysis was performed using R base functions (22).

### Digital droplet PCR

Genomic DNA was extracted from snap-frozen cell PDAC tissue or cell pellets using QIAGEN DNeasy Blood and Tissue Kit. Reactions were performed with digital droplet PCR (ddPCR) Supermix and primers and probes (Bio-Rad) listed in the following text. ddPCR was carried out according to Bio-Rad’s protocol. Droplets were generated on Bio-Rad’s QX200 with droplet generation oil, subjected to amplification (95°C 10 minutes, 94°C 30 seconds, 59°C 1 minute, repeated 40×, 98°C 10 minutes, 8°C hold), and read on Bio-Rad’s QX200 Droplet Reader running QuantaSoft software. Primer sequences are as follows: KRAS-G12D forward: CTG​CTG​AAA​ATG​ACT​GAG​TA, reverse: ATT​AGC​TGT​ATC​GTC​AAG​G, and probe: TGGAGCTGATGGCGT with fluoroscein amidite (FAM) fluorophore; KRAS-WT forward: CTG​CTG​AAA​ATG​ACT​GAG​TA, reverse: ATT​AGC​TGT​ATC​GTC​AAG​G, and probe: TGGAGCTGGTGGCG with hexachlorofluoroscein (HEX) fluorophore.

### Statistical analyses

Statistical analyses were performed using Microsoft Excel 2016 or GraphPad Prism (version 9.1). For endpoint analyses, statistical comparisons were performed using the Mantel–Cox (log-rank) test. Box plots depict the interquartile range (IQR), the median is indicated by the central line, and whiskers indicate minimum and maximum values. Error bars and the relevant statistical test are indicated in each figure legend.

### Data availability

The transcriptional data generated in this study are publicly available in Gene Expression Omnibus (https://www.ncbi.nlm.nih.gov/geo), with accession number GSE277392. All other raw data are available upon request from the corresponding author.

## Results

### Loss of WT *Kras* facilitates pancreatic tumor initiation by oncogenic KRAS

**Figure 1.**
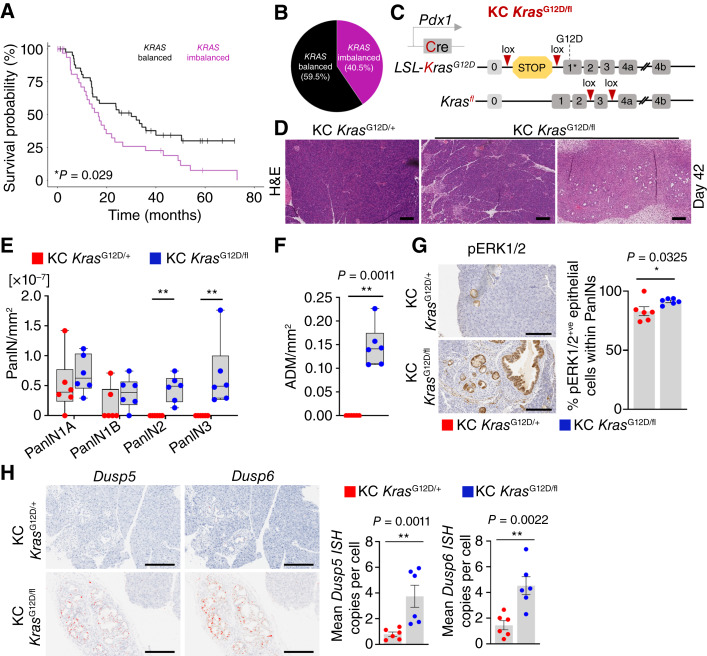
Loss of WT *Kras* increases PanIN formation in the presence of oncogenic *Kras*. **A,** Kaplan–Meier survival curve for human patients with PDAC with *KRAS* alleles balanced and *KRAS* alleles imbalanced. KRAS balanced, *n* = 47; KRAS imbalanced, *n* = 32. *, *P* = 0.029, log-rank (Mantel–Cox) test. **B,** Proportion of human patients with pancreatic cancer with *KRAS* alleles balanced (59.5%) and *KRAS* alleles imbalanced (40.5%), from **A**. **C,** Schematic representing generation of KC *Kras*^G12D/fl^ mice. **D,** Representative hematoxylin and eosin (H&E) images of pancreata from KC *Kras*^G12D/+^ and KC *Kras*^G12D/fl^ mice at 42 days of age, representative of six mice per group. Scale bar, 200 μm. **E,** Quantification and grading of PanINs from KC *Kras*^G12D/+^ and KC *Kras*^G12D/fl^ mice at 42 days of age from one whole hematoxylin and eosin section per mouse (*n* = 6 per group), represented by **D**. Boxes depict the IQR, the central line indicates the median, and whiskers indicate minimum/maximum values. **, *P* < 0.01; one-way Mann–Whitney *U* test. **F,** Quantification of the area of ADM per mm^2^ pancreas over one whole hematoxylin and eosin section from KC *Kras*^G12D/+^ and KC *Kras*^G12D/fl^ mice at 42 days of age (*n* = 6 per group). Boxes depict the IQR, the central line indicates the median, and whiskers indicate minimum/maximum values. **, *P* < 0.0011; one-way Mann–Whitney *U* test. **G,** Left, representative IHC images of pERK1/2 of pancreata from KC *Kras*^G12D/+^ and KC *Kras*^G12D/fl^ mice at 42 days of age. Representative of six mice per group. Scale bar, 200 μm. Right, bar graphs showing quantification of pERK1/2-positive cells of the pancreatic epithelium of KC *Kras*^G12D/+^ and KC *Kras*^G12D/fl^ mice sampled at day 42 (KC *Kras*^G12D/+^, *n* = 6; KC *Kras*^G12D/fl^, *n* = 6). Data are the mean ± SEM. *, *P* = 0.0325; one-way Mann–Whitney *U* test. **H,** Left, representative images of ISH of *Dusp5* and *Dusp6* of pancreata from KC *Kras*^G12D/+^ and KC *Kras*^G12D/fl^ mice at 42 days of age. Representative of six mice per group. Scale bar, 200 μm. Right, quantification of *Dusp5* and *Dusp6 ISH* staining of pancreatic lesions (PanINs) of pancreata from KC *Kras*^G12D/+^ and KC *Kras*^G12D/fl^ mice at 42 days of age (KC *Kras*^G12D/+^, *n* = 6; KC *Kras*^G12D/fl^, *n* = 6). Data are the mean ± SEM. **, *P* = 0.0011 (*Dusp5*); **, *P* = 0.0022 (*Dusp6*); one-way Mann–Whitney *U* test. Cre, Cre recombinase; loxP, Cre-loxP recombination site.

In patients with pancreatic cancer, the occurrence of allelic imbalance at the *KRAS* locus is associated with reduced survival (Fig. 1A; ref. 3) and early progression, as previously reported (2, 5). In the Bailey and colleagues (13) study, the estimated proportion of patients with *KRAS* allelic imbalance is 40.5% (Fig. 1B). Previous work from the Rad group had shown high propensity for allelic imbalances of *KRAS* in human PDAC and strong indication for such imbalances in PanIN progression (5). Nonetheless, analysis of large-scale allelic imbalances of this nature can be confounded by the impact of overall ploidy in the sample cohort (23). To better understand the impact of *Kras* imbalance *in vivo*, we assessed the impact of *Kras* LOH upon both the development of benign pancreatic precursor lesions, known as PanIN, and ultimately the development of pancreatic cancer.

To this end, we modeled precancerous pancreatic lesions and development of pancreatic cancer by targeting mice developing pancreas with a conditional oncogenic *Kras*^LSL-G12D^ allele regulated by a constitutively expressed Cre recombinase, under the control of the *Pdx1* promoter, commonly referred to as *Pdx1*-Cre *Kras*^G12D/+^ (henceforth referred to as KC *Kras*^G12D/+^; ref. 15). Histologically, PanIN formation in the pancreata of these mice can be readily observed from around 10 weeks of age, with low penetrance of PDAC development observed at longer latencies of 6 to 24 months (14, 24). Given that this process is driven by mutant KRAS, we initially sought to determine whether it could be modified by deletion of the WT *Kras* counterpart. To model this, we interbred *Pdx1*-Cre *Kras*^G12D/+^ mice with mice harboring the conditional *Kras*^fl^ allele, thus generating *Pdx1*-Cre *Kras*^G12D/fl^ mice (henceforth referred to as KC *Kras*^G12D/fl^; Fig. 1C).

First, we performed a histologic quantification of the number and stage of precancerous PanIN found in the pancreata of KC *Kras*^G12D/+^ and KC *Kras*^G12D/fl^ mice at 42 days of age. Strikingly, an increase in both number and grade of PanINs, along with extensive nonprogressed yet unresolved acinar–ductal metaplasia (ADM), was observed in the pancreata of KC *Kras*^G12D/fl^ mice when compared with KC *Kras*^G12D/+^ mice (Fig. 1D–E).

It is widely known that expression of oncogenic KRAS activates cellular growth arrest through downstream signaling of tumor-suppressor pathways (25). An equivalent nuclear accumulation of p53 and p21 was observed in the PanIN epithelium of both pancreata of KC *Kras*^G12D/fl^ and KC *Kras*^G12D/+^ mice sampled at the 42-day timepoint (Supplementary Fig. S1A). Moreover, few Ki67-positive cells were observed in the epithelium of PanINs in either model. It was notable that more Ki67-positive cells were detected within the pancreata of KC *Kras*^G12D/fl^ mice when compared with KC *Kras*^G12D/+^ mice; however, these were found within the stromal and inflammatory components of the lesions (Supplementary Fig. S1A).

Activated oncogenic KRAS signaling in PDAC development is directly associated with activation of the downstream MAPK pathway (26). PanINs in both KC *Kras*^G12D/fl^ and KC *Kras*^G12D/+^ models were positive for pERK1/2, indicative of activated MAPK signaling (Fig. 1G). In addition, we analyzed the expression of two well-characterized MAPK-dependent transcripts by ISH (RNA ISH), namely, *Dusp5* and *Dusp6,* in pancreata from KC *Kras*^G12D/fl^ and KC *Kras*^G12D/+^ mice sampled at 42 days. This showed higher expression of both *Dusp5* and *Dusp6*, indicative of increased MAPK signaling, in PanINs from KC *Kras*^G12D/fl^ mice when compared with KC *Kras*^G12D/+^ controls (Fig. 1H), suggesting that loss of the WT *Kras* in our model induces MAPK signaling and drives a transcriptional response in the developing lesion.

Accumulation of PanIN and ADM in KC *Kras*^G12D/fl^ mice was accompanied by a fibrotic reaction shown by picrosirius red and tenascin C positivity, markers for collagen/extracellular matrix deposition (Supplementary Fig. S1B; refs. 27, 28). In addition, an increased abundance of αSMA, a marker for activated fibroblasts, was observed surrounding the epithelium of PanINs in KC *Kras*^G12D/fl^ compared with KC *Kras*^G12D/+^ mice (Supplementary Fig. S1B). An abundant mucin content in the cytoplasm of PanINs of KC *Kras*^G12D/fl^ and KC *Kras*^G12D/+^ mice was demonstrated by alcian blue/periodic acid-Schiff (AB/Pas), a mucin-specific stain.

### Early treatment intervention with MEK1/2 inhibition results in reduction of pancreatic neoplasia in KC mice deficient for WT *Kras*

**Figure 2.**
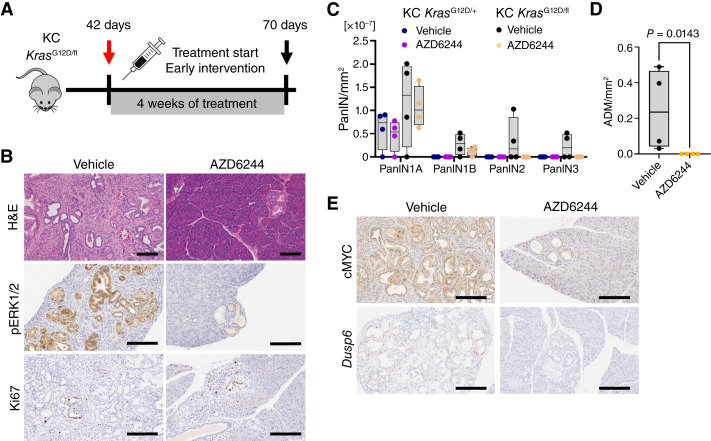
Early intervention with AZD6244 reduces PanIN burden in *Kras*^G12D/fl^ mice. **A,** Experimental schematic. KC *Kras*^G12D/fl^ were treated from day 42 for 28 days with vehicle or AZD6244 and sampled at day 70. **B,** Representative hematoxylin and eosin (H&E), pERK1/2, and Ki67 IHC images from pancreata of KC *Kras*^G12D/fl^ mice at 70 days of age following treatment with vehicle or AZD6244 as indicated for 28 days. Representative of four mice per group. Scale bar, 200 μm. **C,** Quantification and grading of PanINs from pancreata of KC *Kras*^G12D/+^ and KC *Kras*^G12D/fl^ mice treated with vehicle or AZD6244 from 42 to 70 days of age, scored from whole hematoxylin and eosin sections (*n* = 4 per group). Boxes depict the IQR, the central line indicates the median, and whiskers indicate minimum/maximum values. **D,** Quantification of the area of ADM per mm^2^ pancreas over one whole hematoxyly and eosin section from pancreata of KC *Kras*^G12D/fl^ mice treated as indicated from day 42 for 28 days (*n* = 4 per group). Boxes depict the IQR, the central line indicates the median, and whiskers indicate minimum/maximum values. Comparison by one-way Mann–Whitney *U* test. **E,** Representative images of c-MYC IHC and *Dusp6* ISH of KC *Kras*^G12D/fl^ mice treated with vehicle or AZD6244 as indicated from day 42 for 28 days. Representative of four mice per group. Scale bar, 200 μm.

Given the increased PanIN burden and MAPK signaling in the pancreata of KC *Kras*^G12D/fl^ mice, we determined whether these PanIN lesions were sensitive to therapeutic targeting of the MAPK pathway, as has previously been reported in KC *Kras*^G12D/+^ mice (29, 30). To this end, an early intervention study was performed using a clinically relevant inhibitor of the MAPK pathway intermediate kinases, MEK1/2 (AZD6244/selumetinib). As KC *Kras*^G12D/fl^ mice developed substantial PanIN burden by 42 days (Fig. 1D–F), the mice were treated with AZD6244 (25 mg kg^−1^, twice daily by oral gavage) from 42 days of age and sampled at a 70-day timepoint (Fig. 2A). Strikingly, the pancreata of these AZD6244-treated KC *Kras*^G12D/fl^ mice exhibited an apparent reversion of the previously described PanIN burden, with a profoundly reduced area of ADM when compared with vehicle-treated mice and robust loss of pERK1/2 levels and reduced *Dusp6* expression (Fig. 2B–D). Treatment with AZD6244 in KC *Kras*^G12D/+^ had no impact upon PanIN burden, albeit from a starting point of significantly lower PanIN burden than KC *Kras*^G12D/fl^ (Fig. 2C). No change was observed in proliferation in PanINs following AZD6244 treatment compared with control mice (Fig. 2B). Treatment with MEK1/2 inhibitor also resulted in a dramatic decrease in expression of the key transcriptional regulator cMYC in KC *Kras*^G12D/fl^ mice, concomitant with reduced *Dusp6* expression (Fig. 2E).

To understand whether the reduction in ADM resulting from MEK1/2 inhibition is transient and dependent upon the treatment, we performed the early intervention approach, as described above, but followed with a treatment intervention “break” for 7 days. Mice were treated with vehicle or AZD6244 for 28 days and then received no treatment for a further period of 7 days prior to analysis (Supplementary Fig. S2A). Interestingly, even this short break in MAPK suppression was sufficient to allow a robust induction of ADM in KC *Kras*^G12D/fl^ mice. Indeed, the extent of ADM in AZD6244 treatment/release mice was similar to that of vehicle-treated KC *Kras*^G12D/fl^ mice (Supplementary Fig. S2B and S2C). These data support the hypothesis that allelic imbalance at the *Kras* locus with oncogenic *Kras*^G12D^ mutation results in substantial initiation of early pancreatic lesions, primarily in a MAPK-dependent manner.

Our data above suggest that loss of WT KRAS in the context of an oncogenic KRAS^G12D^ mutant can facilitate early tumor initiation, in concordance with prior studies suggesting that under certain circumstances, WT KRAS can act as a tumor suppressor (31) and that loss of WT KRAS can drive progression (32). Therefore, we next questioned whether WT KRAS could act as a tumor suppressor and whether loss of WT KRAS drives progression to pancreatic cancer. We compared the histologic appearance of pancreata from KC *Kras*^G12D/fl^ and KC *Kras*^G12D/+^ mice sampled at various points throughout their lifespan due to common, genotype-related but nonpancreatic phenotypes. Although the pancreata of KC *Kras*^G12D/fl^ mice seemed macroscopically normal, histologically, they were characterized by substantial PanIN burden and the presence of extensive ADM, albeit in the absence of PDAC. Indeed, comparison of KC *Kras*^G12D/fl^ and KC *Kras*^G12D/+^ mice at various points (day 28, day 42, day 160, and day 200) suggested that KC *Kras*^G12D/fl^ mice had substantial and early development of PanIN, ADM, and associated fibrosis (Supplementary Fig. S2D). The rarity of PDAC development in KC *Kras*^G12D/fl^ mice, particularly in the context of dramatically increased PanIN burden, indicates that loss of WT *Kras* facilitates initiation and early lesion formation in the presence of an oncogenic *Kras*^G12D^ mutation but is not sufficient for tumor progression. Thus, in the pancreas, WT *Kras* does not act as a conventional tumor suppressor.

### Loss of WT *Kras* promotes oncogenic *Kras*-driven pancreatic tumorigenesis via increased MAPK signaling

Having observed that loss of WT KRAS in the context of mutated *Kras*^G12D^ was not sufficient to drive PDAC in isolation, we next determined whether additional mutations in other canonical tumor suppressors might allow escape from the observed growth arrest–like phenotype and drive more penetrant pancreatic cancer. We chose to mutate P53 as this was expressed in PanINs of *Kras*^G12D/fl^ mice and its mutation is known to be enriched in human pancreatic cancer and allow senescence escape and drive tumor progression. Therefore, we interbred both KC *Kras*^G12D/fl^ and KC *Kras*^G12D/+^ mice with a mouse carrying an oncogenic mutant *Trp53*^R172H/+^ allele, which encodes a dominant-negative p53, equivalent to one commonly found in human pancreatic cancers, thus generating Pdx1-Cre *Kras*^G12D/fl^*Trp53*^R172H/+^ mice (henceforth referred to as KPC *Kras*^G12D/fl^). In this experimental setting, KPC *Kras*^G12D/fl^ and the equivalent control mice, *Pdx1*-Cre *Kras*^G12D/+^*Trp53*^R172H/+^ (henceforth referred to as KPC *Kras*^G12D/+^), were aged to a clinical endpoint, defined by the onset of clinical signs associated with advanced PDAC.

**Figure 3.**
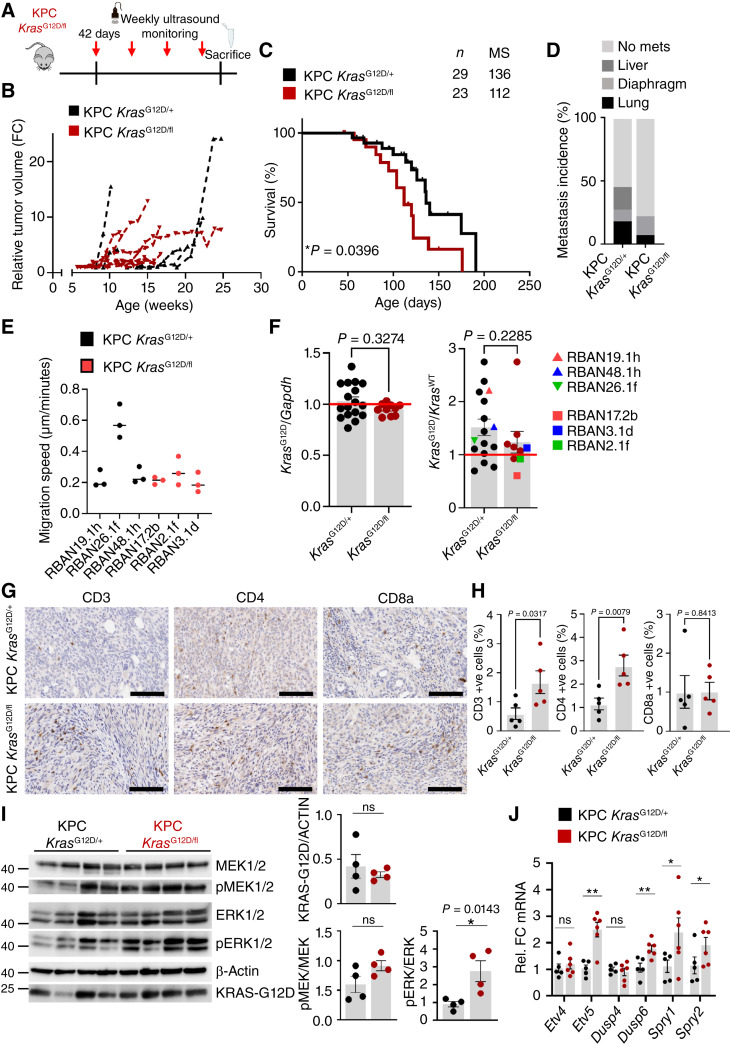
Loss of WT KRAS induces increased MAPK signaling in KPC *Kras*^G12D/fl^. **A,** Experimental schematic representing mice imaged once weekly by ultrasound from 6 weeks of age to clinical endpoint to follow tumor growth over time. **B,** Tumor volume relative to that of tumor at initial detection from KPC *Kras*^G12D/+^ and KPC *Kras*^G12D/fl^ mice aged to clinical endpoint and measured once weekly by ultrasound imaging from 6 weeks of age. KPC *Kras*^G12D/fl^, *n* = 8; KPC *Kras*^G12D/+^, *n* = 10. FC, fold change. **C,** Kaplan–Meier survival curves for KPC *Kras*^G12D/+^ and KPC *Kras*^G12D/fl^ mice aged to clinical endpoint. KPC *Kras*^G12D/fl^, *n* = 29; KPC *Kras*^G12D/+^, *n* = 23. *, *P* = 0.0396 log-rank (Mantel–Cox) test. Mice were censored when reaching clinical endpoint not associated with PDAC burden, such as primary tumors, which arose from other tissue, mostly mucocutaneous papillomas or lymphomas (typically thymic) but also gastric and lung tumors. MS, median survival. **D,** Incidence of metastasis (%) in KPC *Kras*^G12D/+^ and KPC *Kras*^G12D/fl^ mice. KPC *Kras*^G12D/fl^, *n* = 27; KPC *Kras*^G12D/+^, *n* = 20. mets, metastasis. **E,** Migration speed of established cell lines derived from pancreatic cancers arising in KPC *Kras*^G12D/+^ and KPC *Kras*^G12D/fl^ mice. **F,** Relative probe intensity in ddPCR from tumors arising in KPC *Kras*^G12D/+^ (*n* = 16) and KPC *Kras*^G12D/fl^ (*n* = 9) mice at clinical endpoint, comparing *Kras*^G12D^ to *Kras*^WT^ and *Kras*^G12D^ to *Gapdh* probe sets. Tumors from which cell lines analyzed in **I** were generated are highlighted in this analysis. **G,** Representative IHC images of CD3, CD4, and CD8a from PDAC arising in KPC *Kras*^G12D/+^ and KPC *Kras*^G12D/fl^ mice taken at clinical endpoint. Representative of five mice per group. Scale bar, 200 μm. **H,** Bar graphs showing quantification of proportion of CD3-, CD4-, and CD8a-positive cells in PDAC tissue from KPC *Kras*^G12D/+^ and KPC *Kras*^G12D/fl^ mice sampled at clinical endpoint (KPC *Kras*^G12D/+^, *n* = 5; KPC *Kras*^G12D/fl^, *n* = 5). Data are the mean ± SEM, represented in **G**. **I,** Immunoblotting of pMEK1/2, MEK1/2, pERK1/2, ERK1/2, and KrasG12D of KPC *Kras*^G12D/+^ and KPC *Kras*^G12D/fl^ PDAC tissue lysates generated from mice at clinical endpoint. β-Actin was used as a loading control. Each lane represents PDAC tissue from an individual mouse of the indicated genotype. Bar graph shows quantification of pMEK1/2 levels, normalized to MEK1/2; pERK1/2 levels, normalized to ERK1/2; and KRAS-G12D levels, normalized to β-actin. Data represent the mean ± SEM. *, *P* = 0.0143; one-way Mann–Whitney *U* test. **J,** qRT-PCR analysis of *Etv4, Etv5, Dusp4, Dusp6, Spry1,* and *Spry2* in KPC *Kras*^G12D/+^ and KPC *Kras*^G12D/fl^ PDAC tissue. Transcript levels were normalized to *Gapdh* (KPC *Kras*^G12D/+^, *n* = 5; KPC *Kras*^G12D/fl^, *n* = 6). Data represent the mean ± SEM. *P* = 0.2 (*Etv4*); **, *P* = 0.0087 (*Etv5*); *P* = 0.4 (*Dusp4*); **, *P* = 0.0087 (*Dusp6*); *, *P* = 0.0152 (*Spry1*); and *, *P* = 0.0411 (*Spry2*); ns, nonsignificant; one-way Mann–Whitney *U* test. Rel., relative.

Given that a profound lesion initiation phenotype was observed in KC *Kras*^G12D/fl^ mice, tumor development was monitored in KPC *Kras*^G12D/fl^ and KPC *Kras*^G12D/+^ mice from 42 days of age using high-resolution ultrasound imaging (Fig. 3A). Pancreatic cancer was detected in KPC *Kras*^G12D/fl^ mice via palpation, with confirmation by ultrasound imaging at a median of 50 days of age, in contrast to KPC *Kras*^G12D/+^ controls, in which tumor formation was confirmed at a median of 107 days, indicating that loss of WT *Kras* facilitates tumor initiation (Fig. 3B; Supplementary Fig. S3A and S3C). Despite the earlier tumor initiation, we found that KPC *Kras*^G12D/fl^ tumors grew at a comparably slower rate after initial detection of tumor formation, resulting in a marked, albeit nonsignificant, extension in time from tumor onset to experimental endpoint in KPC *Kras*^G12D/fl^ mice (Fig. 3B; Supplementary Fig. S3C). Nonetheless, overall median pancreatic tumor–free survival of KPC *Kras*^G12D/fl^ mice was significantly shorter (median of 112 days) than that of KPC *Kras*^G12D/+^ control mice (median of 136 days; *, *P* = 0.0396, Kaplan–Meier; Fig. 3C). Importantly, KPC *Kras*^G12D/fl^ mice developed fewer metastases when compared with the control KPC *Kras*^G12D/+^ cohort (Fig. 3D). This suppressed metastasis suggests that KPC *Kras*^G12D/fl^ tumor cells may be less aggressive/migratory than those arising in the control cohort. However, analysis of the migration of cells derived from KPC *Kras*^G12D/fl^ and KPC *Kras*^G12D/+^ tumors indicated little difference *in vitro*. This said, it is intriguing to note that of the KPC *Kras*^G12D/+^ derived lines tested, those with similar migratory behavior to KPC *Kras*^G12D/fl^ exhibited allelic imbalance at the *Kras* locus, as determined by ddPCR (Fig. 3E and F).

The analysis of allelic frequencies in tumors arising from each model was carried out via ddPCR both to confirm allelic status in KPC *Kras*^G12D/fl^ and to determine whether sporadic LOH events resulted in allelic imbalance in KPC *Kras*^G12D/+^ control mice. This analysis indicated that there is a tendency toward an increase in the allelic frequency of *Kras*^G12D^ relative to the WT allele in KPC *Kras*^G12D/+^ tumors (ratio > 1; Fig. 3F), suggestive of allelic imbalance. Meanwhile, the frequency of the *Kras*^G12D^ relative to a control probe (*Gapdh*) suggests that there are no allelic gains of *Kras*^G12D^ (ratio≈1; Fig. 3F). Cumulatively, these data suggest that some KPC *Kras*^G12D/+^ tumors lost the WT Kras allele akin to the KPC *Kras*^G12D/fl^ tumors by the time the mice reach clinical endpoint. Notably there were no allelic changes identified in the KPC *Kras*^G12D/fl^ model, presumably due to a lack of selective pressure to lose a silenced allele.

In terms of broad histopathologic analysis, there were few qualitative differences between primary pancreatic tumors arising in KPC *Kras*^G12D/fl^ when compared with KPC *Kras*^G12D/+^ mice (Supplementary Fig. S3B). Although quantification of BrdU incorporation into tumors at endpoint indicated that KPC *Kras*^G12D/fl^ tumors were no more proliferative than KPC *Kras*^G12D/+^, accumulation of mutant p53 was reduced in KPC *Kras*^G12D/fl^ compared with KPC *Kras*^G12D/+^ tumors (Supplementary Fig. S3D and S3E). In the KPC *Kras*^G12D/fl^ tumors, the irregular tumor glands of PDAC seem to be surrounded by desmoplastic stroma, composed of stromal, inflammatory, and immune cells along with the characteristic extracellular matrix. In contrast to the KC *Kras*^G12D/fl^ mice, the stromal architecture appeared equivalent in KPC *Kras*^G12D/fl^ and KPC *Kras*^G12D/+^ tumors observed by αSMA and picrosirius red staining (Supplementary Fig. S3D and S3E). Quantification of the immune architecture of the tumors indicated an induction of immune infiltrate in KPC *Kras*^G12D/fl^ tumors when compared with controls. More specifically, there was a marked increase in CD3^+ve^ and CD4^+ve^ lymphocyte populations, although this did not seem to translate into increased cytotoxic CD8^+ve^ infiltration (Fig. 3G and H). Nonetheless, it is notable that loss of the WT *Kras* allele seems to be associated with immune infiltration in a setting that is classically considered highly immune-excluded.

Given the apparent discordance between the early tumor initiation phenotypes and subsequent tumor growth, we next sought to better understand the molecular impact that loss of WT *Kras* has in the context of an oncogenic *Kras*^G12D^ mutant in pancreatic tumors *in vivo*. First, we determined whether loss of WT *Kras* altered the proportion of GTP-bound (or “active”) KRAS found in KPC *Kras*^G12D/fl^ and KPC *Kras*^G12D/+^ tumors. Using RAF-RBD pulldowns, we observed no changes in active KRAS, suggesting an equal proportion of active KRAS in KPC *Kras*^G12D/fl^ and KPC *Kras*^G12D/+^ tumors (Supplementary Fig. S4A and S4B).

There was a marked upregulation of the MAPK-dependent transcripts *Dusp5* and *Dusp6* in early pancreatic lesions in the KC *Kras*^G12D/fl^ model. These transcripts encode negative regulators of the MAPK pathway, comprising a well-established feedback mechanism. This suggests an increase in downstream signaling as a result of *Kras* allelic imbalance, and as such is in line with previous research (33). Therefore, we investigated whether loss of the WT *Kras* allele in late-stage murine pancreatic tumors can also alter signaling through downstream effector pathways and activate any associated feedback loops. Tumors arising in KPC *Kras*^G12D/fl^ mice exhibited substantially increased pERK1/2 when compared with control KPC *Kras*^G12D/+^ tumors (Fig. 3I). This pattern contrasts with that seen in early-initiating lesions and suggests a robust upregulation of MAPK signaling in tumors arising in the KPC *Kras*^G12D/fl^ model when compared with KPC *Kras*^G12D/+^ controls. Notably, other common effector pathways downstream of KRAS, such as the PI3K signaling pathway, seemed unchanged (Supplementary Fig. S4C). This induction of MAPK signaling in KPC *Kras*^G12D/fl^ tumors was supported by significantly increased expression of MAPK-dependent transcriptional targets such as *Etv5, Dusp6, Spry1,* and *Spry2* in KPC *Kras*^G12D/fl^ tumors compared with controls (Fig. 3J). In accordance, protein levels of DUSP6 were found to be elevated in KPC *Kras*^G12D/fl^ tumors compared with KPC *Kras*^G12D/+^ mouse tumors (Supplementary Fig. S4C). These data suggest that in the context of a pancreatic tumor, loss of WT *Kras* potentiates oncogenic KRAS-driven MAPK signaling and transcription of downstream target genes.

Several recent studies have highlighted that transcriptional profiling can be a useful tool in understanding and the classification of human PDAC. Given the clear phenotypic differences between the KPC *Kras*^G12D/fl^ and KPC *Kras*^G12D/+^ models (Fig. 3), we sought to determine whether transcriptional profiling of these tumors could be used to stratify and align to previously described human PDAC subtype classifications. Analysis of KPC *Kras*^G12D/fl^ and KPC *Kras*^G12D/+^ tumors using the Bailey classification suggested no significant enrichment for any subtype in both KPC *Kras*^G12D/+^ and KPC *Kras*^G12D/fl^ tumors (Supplementary Fig. S4D; refs. 13, 34). However, gene programs GP6, GP7, and GP8 from the Bailey classification, all of which are associated with immune cell-specific gene expression signatures, were significantly enriched in tumors arising in KPC *Kras*^G12D/fl^ mice (Supplementary Fig. S4E). These immune signatures include gene programs representative of B-cell signaling, antigen presentation, and critically, given apparent infiltration of CD3 and CD4^+ve^ immune populations (Fig. 3G and H) into KPC *Kras*^G12D/fl^ tumors, with T-lymphocyte–associated signaling pathways. Moreover, immune regulatory hallmark gene sets such as “IFNα response” and “IFNγ response,” “inflammatory response,” and “allograft rejection,” all gene programs associated with immune surveillance, were also significantly enriched in KPC *Kras*^G12D/fl^ tumors compared with KPC *Kras*^G12D/+^ tumors (Supplementary Fig. S4F).

### Loss of WT *Kras* sensitizes late-stage pancreatic tumors to MEK1/2 inhibition

**Figure 4.**
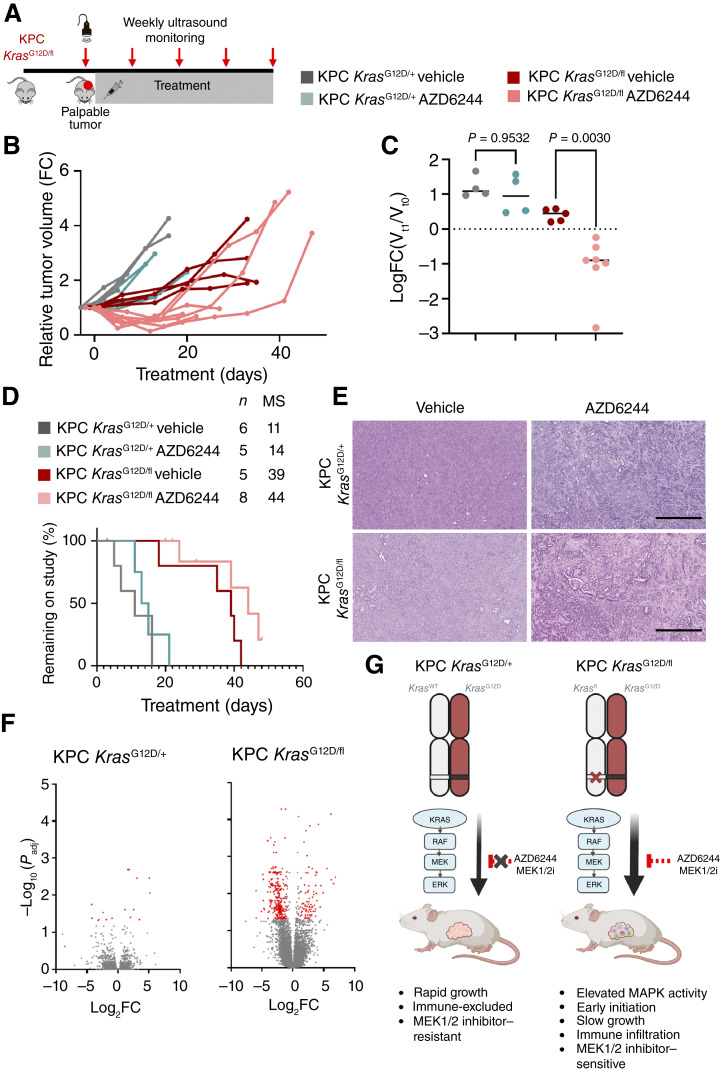
Late-stage intervention with MEK1/2 inhibition improves survival of KPC *Kras*^G12D/fl^ mice. **A,** Experimental schematic. KPC *Kras*^G12D/+^ and KPC *Kras*^G12D/fl^ mice were palpated for tumor burden, with palpable tumor burden confirmed by ultrasound imaging, with mice treated continuously from the following day with either vehicle or AZD6244. Tumor growth was monitored by ultrasound imaging once weekly to clinical endpoint. **B,** Relative volume of pancreatic tumors arising in KPC *Kras*^G12D/+^ and KPC *Kras*^G12D/fl^ mice treated with either vehicle control or AZD6244 from palpable tumor and aged to clinical endpoint. Tumor volume was measured once weekly by high-resolution ultrasound imaging. Each line represents an individual mouse of the indicated genotype and treatment. KPC *Kras*^G12D/fl^ vehicle, *n* = 6; KPC *Kras*^G12D/fl^ AZD6244, *n* = 7; KPC *Kras*^G12D/+^ vehicle, *n* = 6; KPC *Kras*^G12D/+^ AZD6244, *n* = 4. FC, fold change. **C,** Change of tumor volume (mm^3^) of KPC *Kras*^G12D/+^ and KPC *Kras*^G12D/fl^ mice treated with either vehicle control or AZD6244 from palpable tumor between initial ultrasound measurement and a secondary ultrasound measurement (between 6 and 13 days later). Plot represents Log_2_(FC) of tumor volume between first and second measurements for the full treatment cohort when more than one imaging session was possible. KPC *Kras*^G12D/fl^ vehicle, *n* = 5; KPC *Kras*^G12D/fl^ AZD6244, *n* = 7; KPC *Kras*^G12D/+^ vehicle, *n* = 4; KPC *Kras*^G12D/+^ AZD6244, *n* = 4. **D,** Kaplan–Meier survival curves for KPC *Kras*^G12D/+^ and KPC *Kras*^G12D/fl^ mice treated with vehicle or AZD6244, as indicated, from palpable tumor burden and aged to clinical endpoint. KPC *Kras*^G12D/fl^ vehicle, *n* = 5; KPC *Kras*^G12D/fl^ AZD6244, *n* = 8; KPC *Kras*^G12D/+^ vehicle, *n* = 6; KPC *Kras*^G12D/+^ AZD6244, *n* = 5. MS, median survival. **E,** Representative hematoxylin and eosin images of tumors from KPC *Kras*^G12D/+^ and KPC *Kras*^G12D/fl^ mice treated with vehicle or AZD6244, as indicated, from palpable tumor burden and aged until clinical endpoint. Representative of five mice per group. Scale bar, 500 μm. **F,** Left, volcano plot for differentially expressed genes of KPC *Kras*^G12D/+^ tumors treated with vehicle or AZD6244 from palpable tumor burden to clinical endpoint. Right, volcano plot for differentially expressed genes of KPC *Kras*^G12D/fl^ tumors treated with vehicle or AZD6244 from palpable tumor burden to clinical endpoint. Red, significantly altered genes. **G,** Schematic representing relative impact of WT Kras deletion upon tumur outgrowth and therapeutic responses from KPC *Kras*^G12D/+^ and *Kras*^G12D/fl^ comparison.

Given that MAPK signaling was enriched in KPC *Kras*^G12D/fl^ tumors, we next investigated whether MEK1/2 inhibition is effective in the context of an established pancreatic tumor. This is of particular importance as the majority of patients with pancreatic cancer present with late-stage, established, and difficult-to-treat disease. To achieve this, cohorts of KPC *Kras*^G12D/fl^ and KPC *Kras*^G12D/+^ mice with palpable tumors confirmed by ultrasound were enrolled into treatment groups and received AZD6244 or the vehicle control. Tumor growth was monitored once weekly by ultrasound imaging over the course of treatment (Fig. 4A). Consistent with previous reports, we observed that control KPC *Kras*^G12D/+^ tumors were resistant to MEK1/2 inhibition. The AZD6244- or vehicle-treated KPC *Kras*^G12D/+^ mice showed no overall difference in survival, and both had a continuous, rapid, and comparable tumor growth (Fig. 4B–D). Strikingly, ultrasound monitoring and measurement of tumors demonstrated significant tumor shrinkage in KPC *Kras*^G12D/fl^ mice following enrollment on AZD6244 treatment but not following vehicle treatment (Fig. 4B and C). This tumor shrinkage was detectable within the first 7 days of treatment, and the tumor volume typically remained stable or further shrank for up to 21 days after beginning treatment. After this 21-day period, therapeutic resistance was acquired, and tumors progressed and grew rapidly (Fig. 4B). This translated into an extension of survival in KPC *Kras*^G12D/fl^ mice treated with AZD6244 compared with vehicle-treated control mice (Fig. 4D). There did not seem to qualitative/gross histopathologic differences in response to MAPK inhibition with AZD6244 between KPC *Kras*^G12D/fl^ and KPC *Kras*^G12D/+^ tumors (Fig. 4E).

To gain further insight into mechanisms that drive sensitivity to AZD6244 in KPC *Kras*^G12D/fl^ tumors, and thus potentially identify targets or pathways that might sensitize the control KPC *Kras*^G12D/+^ tumors, we performed transcriptomic analysis of tumors from KPC *Kras*^G12D/fl^ or KPC *Kras*^G12D/+^ cohorts treated with either AZD6244 or vehicle from tumor detection. In the AZD6244 treatment–resistant KPC *Kras*^G12D/+^ tumors, AZD6244 treatment induced very few significantly differentially regulated genes groups (Fig. 4F). In contrast, in KPC *Kras*^G12D/fl^ tumors, which were sensitive to AZD6244 treatment (MEK1/2 inhibition), there were a substantially higher number of differentially regulated genes in AZD6244-treated versus vehicle-treated groups (Fig. 4F). Transcriptional analysis revealed elevated MYC target gene expression in MEK1/2 inhibitor–treated KPC *Kras*^G12D/fl^ mice at clinical endpoint, suggestive of activated proliferative programs associated with acquired resistance to therapy (Supplementary Fig. S6A). Transcriptional subtype classification of KPC *Kras*^G12D/+^- or KPC *Kras*^G12D/fl^-derived tumors indicated that treatment with AZD6244 had no impact upon subtype in KPC *Kras*^G12D/+^ tumors, further highlighting the robust resistance to MEK1/2 inhibition in this setting, whereas enrichment for the immunogenic subtype in KPC *Kras*^G12D/fl^ tumors was reversed by treatment with AZD6244 (Supplementary Fig. S5A). This was associated with an apparent loss of enrichment of the immune signaling–associated gene programs GP6, GP7, and GP8 following AZD6244 treatment in this model (Supplementary Fig. S5B) and negative enrichment for immune-associated hallmark gene sets in KPC *Kras*^G12D/fl^ tumors treated with AZD6244 compared with their vehicle-treated counterparts (Supplementary Fig. S6B). The suppression of immune signaling–associated gene programs and immune hallmark gene sets did not translate into a detectable reduction of lymphocyte infiltration into KPC *Kras*^G12D/fl^ tumors at this timepoint (Supplementary Fig. S6C and S6D).

The robust therapeutic response elicited by AZD6244 suggests that KPC *Kras*^G12D/fl^ tumors have increased dependency upon MAPK signaling and suggests that a therapeutic opportunity may exist in patients with allelic imbalance at the *KRAS* locus for MAPK-targeting therapeutic approaches. The differential response to AZD6244 observed in KPC *Kras*^G12D/fl^ and KPC *Kras*^G12D/+^ tumors further supports the hypothesis that loss of the WT *Kras* allele profoundly affects the oncogenic signaling within pancreatic tumors and that the presence of a WT *Kras* allele can contribute to therapeutic resistance in *Kras* mutant tumors.

## Discussion

For many years, research has been focused on understanding oncogenic driver genes and their functional impact in cancer. Recent studies provide evidence that not only oncogenic *RAS* mutations but also the relative oncogene gene dosage due to allelic imbalance and copy-number variations are modulators of oncogenic signaling output, tumor development, and incidence of metastasis (5, 10, 11). Notably, there has been growing evidence that *RAS* relative gene dosage modulates response to therapy (11).

Our data provide evidence that loss of WT *Kras* plays a crucial role in PDAC biology and phenotypic diversification. We developed a GEMM of *Kras* LOH in pancreatic cancer. This study shows that pancreatic cancer initiation was facilitated in WT *Kras*–depleted mice in the context of oncogenic *Kras*, observed by increased numbers and more advanced precursor lesions (PanIN) and extensive ADM in the pancreas of WT *Kras*–deficient *Kras* mutant mice. Loss of WT *KRAS* in the presence of mutant *KRAS*^G12D^ has been shown to induce MAPK signaling, as observed by transcriptional activation of MAPK regulators. We show that depletion of WT *Kras* in *Kras* mutant mice facilitates tumorigenesis in the pancreas yet was not sufficient to drive progression to PDAC, suggesting that WT KRAS acts to restrain mutant KRAS signaling but does not act as tumor suppressor in pancreatic cancer.

Previous studies suggest that the presence of WT RAS modulates response to treatment in oncogenic RAS-driven tumors (11, 33). Whereas WT KRAS–proficient tumors were resistant to MAPK inhibition with a MEK1/2 inhibitor, we observed a robust initial response to treatment in KPC *Kras*^G12D/fl^ tumors in contrast to KPC *Kras*^G12D/+^ controls. Whereas the molecular basis of this differential response is unclear, it is tempting to speculate that loss of the WT allele not only drives increased oncogenic signaling but also removes significant capacity to buffer KRAS/MAPK signaling or indeed regulate responses to extrinsic signals. The corollary of this would be that KPC *Kras*^G12D/+^ cells might therefore be less well-equipped to maintain or buffer intrinsic MAPK signaling in response to targeted inhibition. Moreover, there is growing literature demonstrating specific effector-binding profiles associated with mutant KRAS when compared with WT KRAS, including increased activation of the MAPK pathway potentially rendering monoallelic mutant tumors more sensitive to inhibition (35).

Critically, the tumor shrinkage in KPC *Kras*^G12D/fl^ mice treated with AZD6244 is an important point to highlight, as many patients present clinically with advanced pancreatic cancer that is not suitable for surgical resection, which is the only effective cure. Therefore, even transient therapeutic response that drives tumor shrinkage in a defined subset of patients (patients with *KRAS* LOH) might offer a promising treatment opportunity and potential clinical benefit in the neoadjuvant setting. Indeed, clinical data have established that neoadjuvant treatment in borderline resectable pancreatic cancer can be an effective approach (36, 37).

WT *Kras* deletion had profound effects on the transcriptional landscape of tumors, suggesting phenotypic alteration in tumors deficient for WT *Kras*. A specific enrichment for the immunogenic subtype was observed in the KPC *Kras*^G12D/fl^ tumors, along with upregulation of immune cell–specific gene programs. Given the enrichment for immunogenic gene programs, this may suggest that specific a subset of patients with LOH *Kras* allelic imbalance may benefit from immunotherapy with agents including anti-PD-1/PD-L1, -CTLA4, -TIGIT, -41BB or -LAG3–targeting therapies (38, 39), which should be the subject of further investigation.

In summary, these data demonstrate that even simple allelic imbalances at the *Kras* locus can have a profound impact upon all aspects of the tumorigenic process, encompassing initiation, progression, and therapeutic sensitivity. These data in turn suggest that the presence of a WT copy of *Kras* can act to suppress the impact of oncogenic *Kras*. We have gone on to show that preclinical models featuring allelic imbalance at the *Kras* locus in the context of oncogenic *Kras* may represent a subset of patients with pancreatic cancer in the clinic. Finally, we demonstrate significant therapeutic efficacy of clinically relevant inhibitors of the MAPK pathway in this preclinical setting, hinting at the possibility of clinical efficacy in a substantial subset of patients (Fig. 4G).

Altogether, the data presented in this study highlight that mutant and WT *Kras* allelic status in *Kras*^G12D^-driven pancreatic cancer has important implications for PDAC biology and therapeutic response. Poor response of patients to therapeutic intervention may be explained by mutant and WT *Kras* allelic status. We demonstrate that stratification of patients by *Kras* allelic imbalances (LOH and mutant *Kras* copy number) might aid to identify therapeutic vulnerabilities and lead to more patient-specific treatment modalities and overall contribute to improved treatments responses and patient care.

## Supplementary Material

Supplementary Figure 1 Increased fibrosis in wild-type Kras deficient PanINs.

Supplementary Figure 2 Release from AZD6244 treatment results in rapid acinar to ductal metaplasia in KC KrasG12D/fl.

Supplementary Figure 3 Acceleration of pancreatic tumour initiation after loss of wild-type Kras in KPC KrasG12D/fl mice.

Supplementary Figure 4 Loss of wild-type KRAS does not alter PI3K-AKT signalling.

Supplementary Figure 5 Loss of wild-type KRAS sensitizes KPC KrasG12D/fl tumours to MEK1/2 inhibition.

Supplementary Figure 6 MEK1/2 inhibition in KPC KrasG12D/fl mice with established tumours reverses enrichment of immune response related gene programmes.
