# Supplementary material for: KRAS Loss of Heterozygosity Promotes MAPK-Dependent Pancreatic Ductal Adenocarcinoma Initiation and Induces Therapeutic Sensitivity to MEK Inhibition
Source: Cancer Res. 2024 Oct 16;85(2):251–62. doi: 10.1158/0008-5472.CAN-23-2709 (PMC11733531; doi:10.1158/0008-5472.CAN-23-2709)
Supplement: Supplementary Figure 1 — Increased fibrosis in wild-type Kras deficient PanINs. [file can-23-2709_supplementary_figure_1_suppsf1.pdf]

Suppl Figure 1

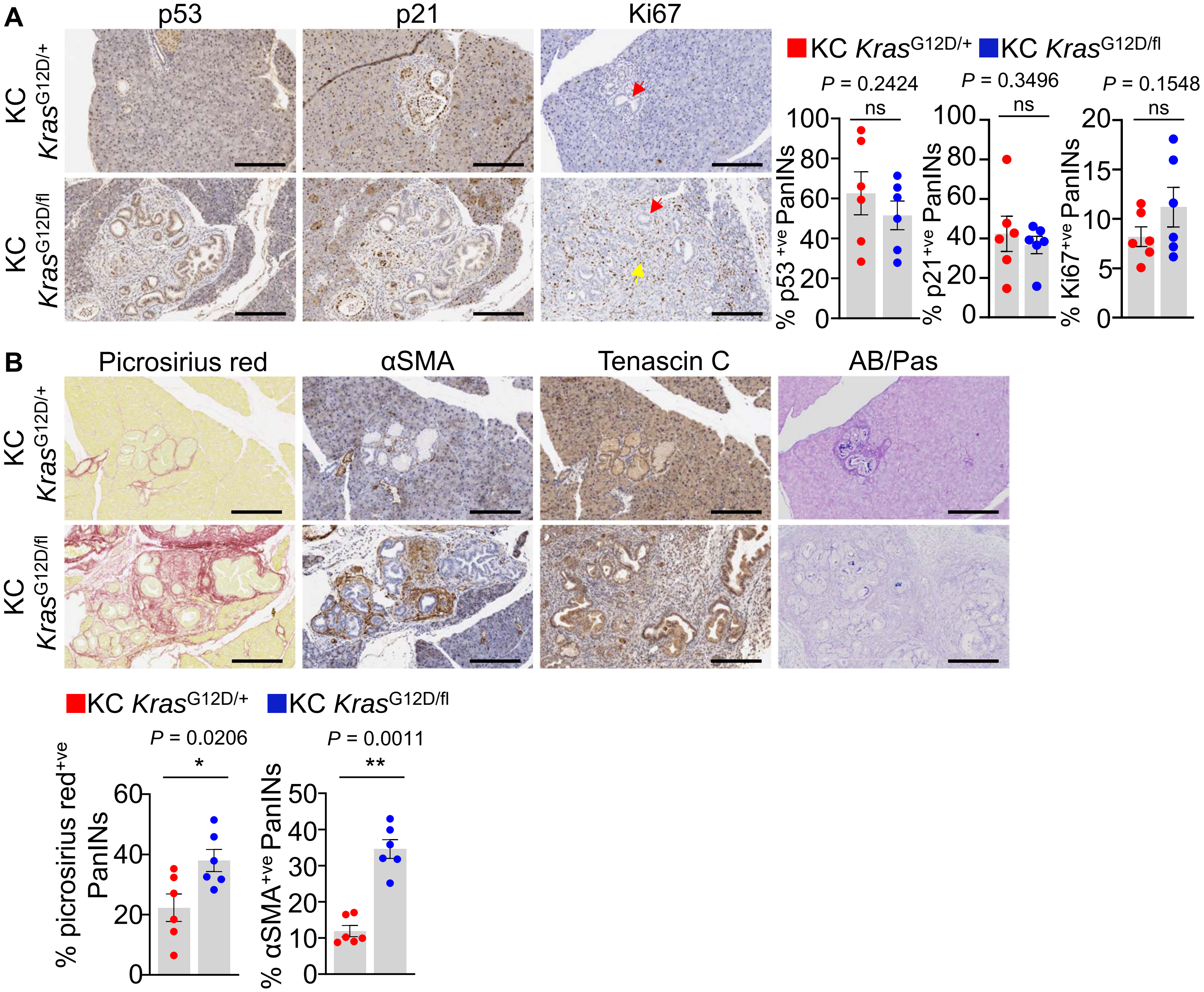

Supplementary Figure 1: Increased fibrosis in wild-type *Kras* deficient PanINs. A) Left: Representative immunohistochemistry images of p53, p21 and Ki67 staining from pancreata of KC *Kras*$^{G12D/+}$ and KC *Kras*$^{G12D/fl}$ mice at 42 days of age. Red arrows indicate PanIN lesions (epithelial) and yellow arrow indicates Ki67 positive cells in regions of acinar-ductal metaplasia. Representative of six mice per group. Scale bar 200 μm. Right: Quantification of p53, p21 and Ki67 positive cells in pancreatic lesions (PanINs) from pancreata of KC *Kras*$^{G12D/+}$ and KC *Kras*$^{G12D/fl}$ mice at 42 days of age (KC *Kras*$^{G12D/+}$, n = 6; KC *Kras*$^{G12D/fl}$, n = 6). Data are mean ± s.e.m, *P* = 0.2424 (p53), *P* = 0.3496 (p21), *P* = 0.1548 (Ki67), one-way Mann–Whitney U test. B) Top: Representative images of picrosirius red and AB/PAS staining and immunohistochemistry of αSMA and Tenascin C from pancreata of KC *Kras*$^{G12D/+}$ and KC *Kras*$^{G12D/fl}$ mice at 42 days of age. Scale bar 200 μm. Bottom: Quantification of picrosirius red and αSMA positivity (%) of PanIN lesions from pancreata of KC *Kras*$^{G12D/+}$ and KC *Kras*$^{G12D/fl}$ mice at 42 days of age (KC *Kras*$^{G12D/+}$, n = 6; KC *Kras*$^{G12D/fl}$, n = 6). Data are mean ± s.e.m, **P* = 0.0206 (picrosirius red), ***P* = 0.0011 (αSMA), one-way Mann–Whitney U test.
