# Supplementary material for: KRAS Loss of Heterozygosity Promotes MAPK-Dependent Pancreatic Ductal Adenocarcinoma Initiation and Induces Therapeutic Sensitivity to MEK Inhibition
Source: Cancer Res. 2024 Oct 16;85(2):251–62. doi: 10.1158/0008-5472.CAN-23-2709 (PMC11733531; doi:10.1158/0008-5472.CAN-23-2709)
Supplement: Supplementary Figure 2 — Release from AZD6244 treatment results in rapid acinar to ductal metaplasia in KC KrasG12D/fl. [file can-23-2709_supplementary_figure_2_suppsf2.pdf]

Suppl Figure 2

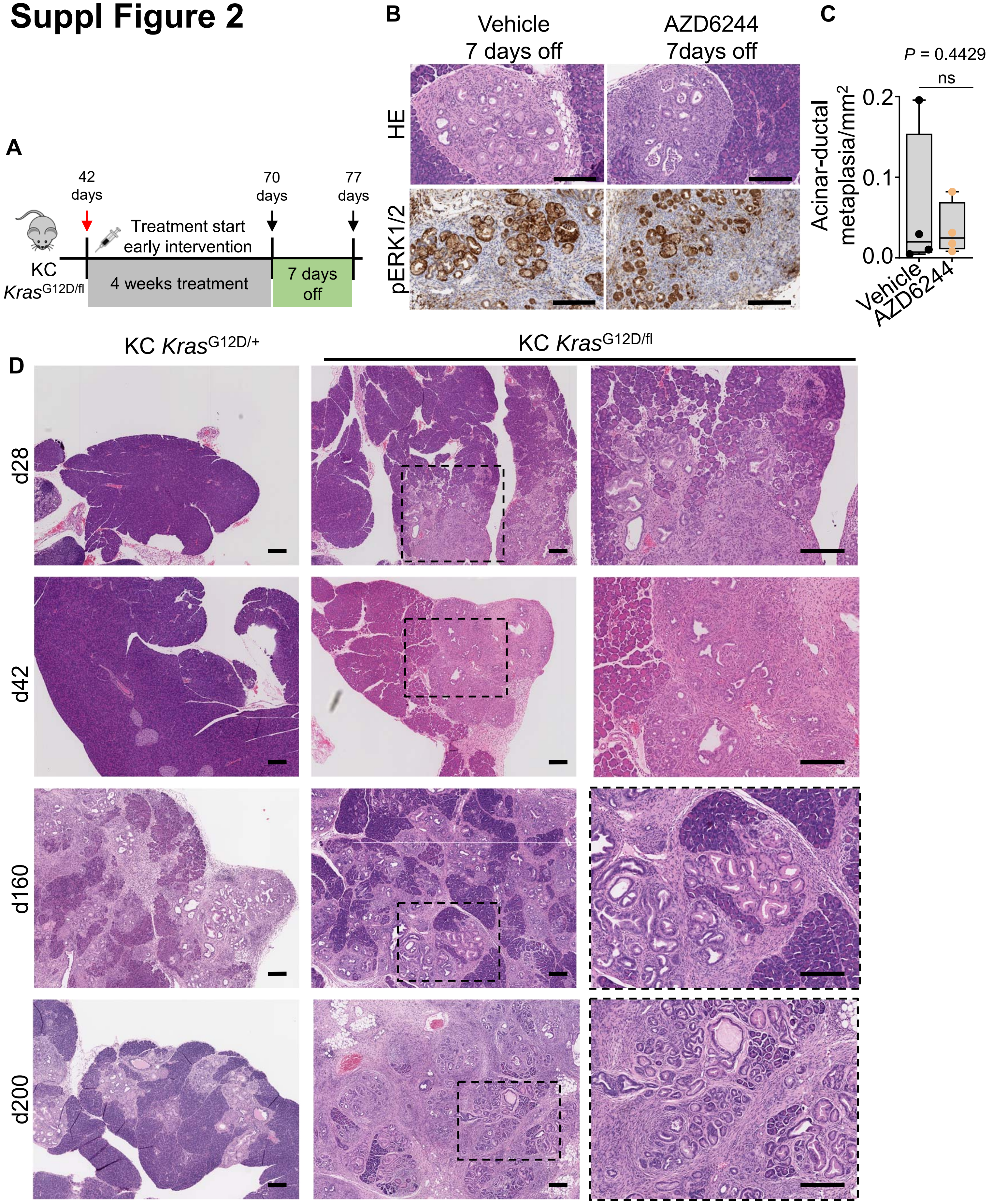

Supplementary Figure 2: Release from AZD6244 treatment results in rapid acinar to ductal metaplasia in KC *Kras*$^{G12D/fl}$. A) Experimental schematic: *Kras*$^{G12D/fl}$ were treated from day 42 for 28 days with vehicle or AZD6244 as indicated prior to a 7-day drug holiday, followed by sampling at day 77. B) Representative H&E and pERK1/2 IHC from KC *Kras*$^{G12D/fl}$ mice at 77 days of age treated as described in (A). Representative of four mice per group. Scale bar 200 µm. C) Quantification of the area of acinar-ductal metaplasia per mm^2 pancreas over one whole H&E section from KC *Kras*$^{G12D/fl}$ mice at 77 days of age treated as described in (A) (n = 4 per group). Boxes depict interquartile range, central line indicates median and whiskers indicate minimum/maximum values. $P = 0.4429$, one-way Mann–Whitney U test. D) H&E images of pancreata from KC *Kras*$^{G12D/+}$ and KC *Kras*$^{G12D/fl}$ mice of indicated timepoints. Dashed boxes show higher magnification. Scale bar 200 µm.
