# Supplementary material for: KRAS Loss of Heterozygosity Promotes MAPK-Dependent Pancreatic Ductal Adenocarcinoma Initiation and Induces Therapeutic Sensitivity to MEK Inhibition
Source: Cancer Res. 2024 Oct 16;85(2):251–62. doi: 10.1158/0008-5472.CAN-23-2709 (PMC11733531; doi:10.1158/0008-5472.CAN-23-2709)
Supplement: Supplementary Figure 3 — Acceleration of pancreatic tumour initiation after loss of wild-type Kras in KPC KrasG12D/fl mice. [file can-23-2709_supplementary_figure_3_suppsf3.pdf]

Suppl Figure 3

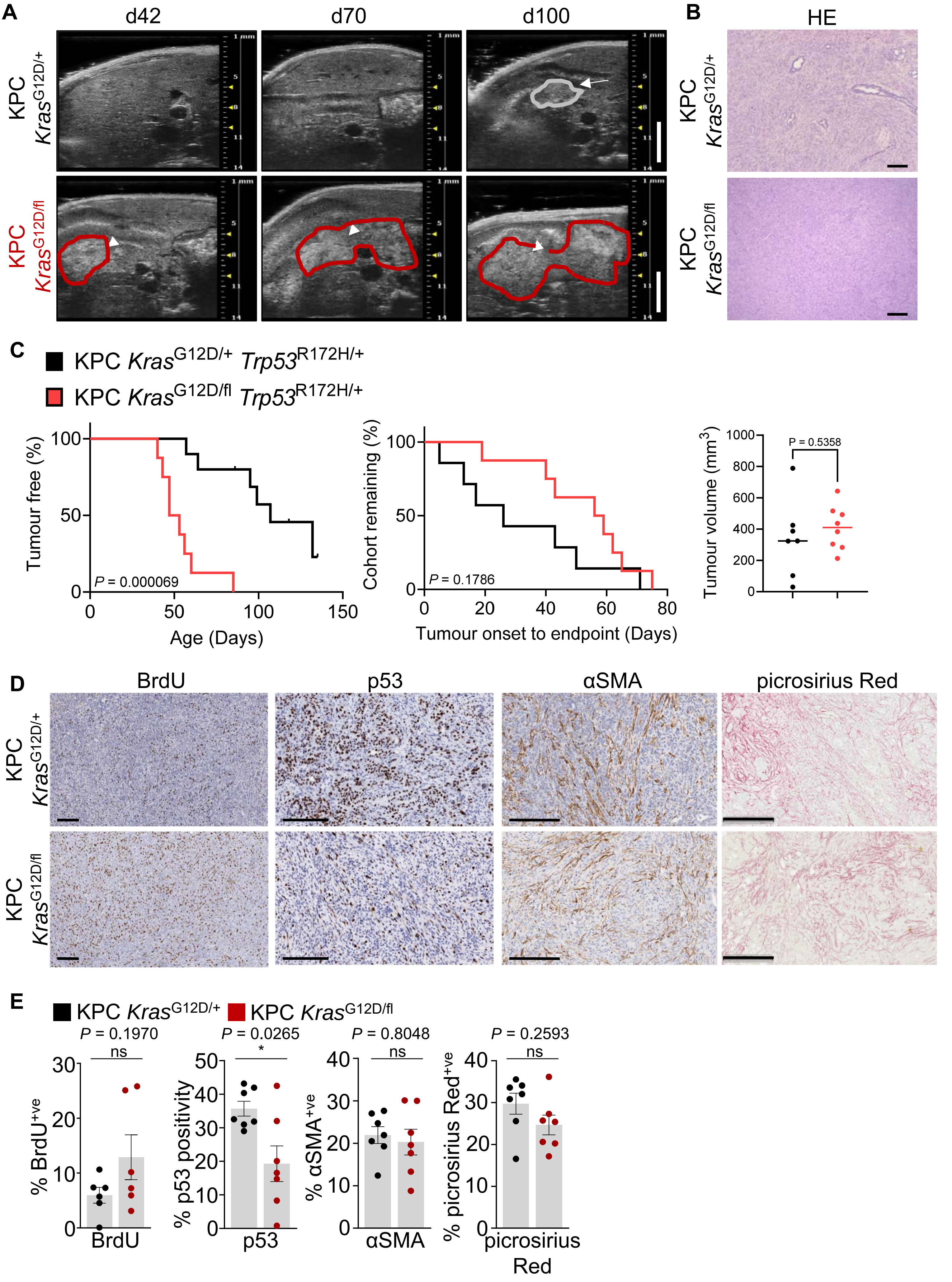

Supplementary Figure 3: Acceleration of pancreatic tumour initiation after loss of wild-type *Kras* in KPC *Kras*$^{G12D/fl}$ mice. A) Representative images of high- resolution ultrasound imaging of KPC *Kras*$^{G12D/+}$ and KPC *Kras*$^{G12D/fl}$ mice at day 42, day 70 and day 100. Arrows and mark up indicate PDAC. Representative of five mice per group. Scale bar 5 mm. B) Representative H&E images of KPC *Kras*$^{G12D/+}$ and KPC *Kras*$^{G12D/fl}$ mice aged to clinical endpoint. Scale bar 200 µm. C) Kaplan Meier analysis of age to tumour mass detectable by high-resolution ultrasound (left) or time from detectable tumour mouse to experimental endpoint (middle), and tumour volume at experimental endpoint as determined by high resolution ultrasound (right). D) Representative images of BrdU, p53, αSMA immunohistochemistry and picrosirius red staining of KPC *Kras*$^{G12D/+}$ and KPC *Kras*$^{G12D/fl}$ mice aged to clinical endpoint, (KPC *Kras*$^{G12D/+}$, n = 7 (n = 6 for BrdU); KPC *Kras*$^{G12D/fl}$, n = 7, (n = 6 for BrdU)). Scale bar 200 µm. E) Quantification of BrdU and p53 positive cells, αSMA and picrosirius red positive area from PDAC of KPC *Kras*$^{G12D/+}$ and KPC *Kras*$^{G12D/fl}$ mice aged to clinical endpoint. (KPC *Kras*$^{G12D/+}$, n = 7, (n = 6 for BrdU); KPC *Kras*$^{G12D/fl}$, n = 7 (n = 6 for BrdU)). Data are mean ± s.e.m, *P* = 0.1970 (BrdU), **P* = 0.0265 (p53), *P* = 0.8048 (αSMA), *P* = 0.2593 (picrosirius red), one-way Mann–Whitney U test.
