# Supplementary material for: KRAS Loss of Heterozygosity Promotes MAPK-Dependent Pancreatic Ductal Adenocarcinoma Initiation and Induces Therapeutic Sensitivity to MEK Inhibition
Source: Cancer Res. 2024 Oct 16;85(2):251–62. doi: 10.1158/0008-5472.CAN-23-2709 (PMC11733531; doi:10.1158/0008-5472.CAN-23-2709)
Supplement: Supplementary Figure 4 — Loss of wild-type KRAS does not alter PI3K-AKT signalling. [file can-23-2709_supplementary_figure_4_suppsf4.pdf]

Suppl Figure 4

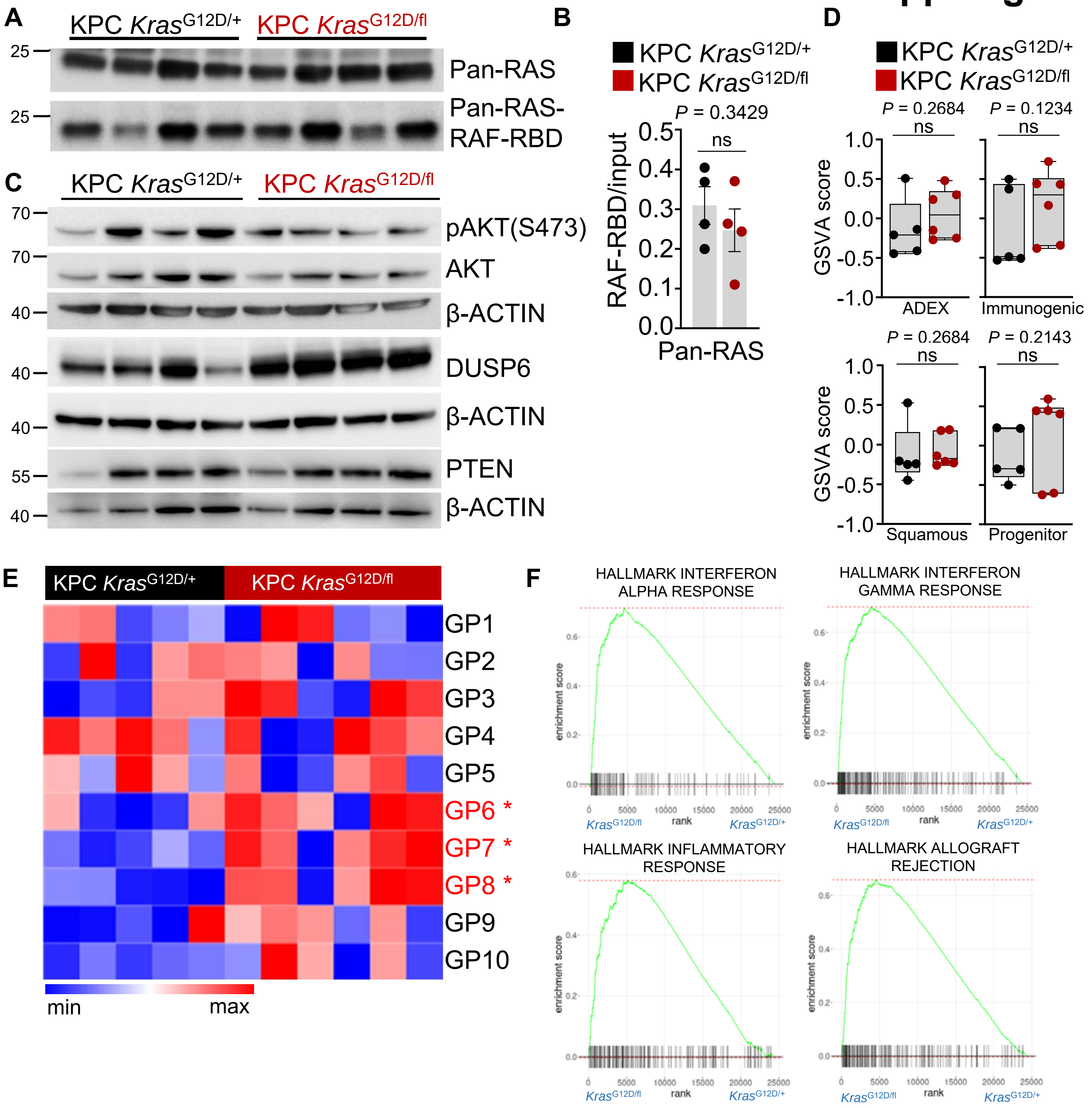

Supplementary Figure 4: Loss of wild-type KRAS does not alter PI3K-AKT signalling. A) RAF-RBD agarose affinity purification assay of KPC *Kras*$^{G12D/+}$ and KPC *Kras*$^{G12D/fl}$ from PDAC tissue of four biological independent samples per condition. Pulldown of RAS-GTP with RAF-RBD agarose beads. Precipitates were immunoblotted using a pan-RAS antibody and input pan-RAS served as loading control. B) Bar graphs representing RAS-GTP activation levels quantified and normalized to pan-RAS loading control (from S4a). N = 4 biological replicates per group, each lane represents snap frozen PDAC tissue lysate generated from individual mice from genotype indicated. Data represent mean ± s.e.m., P = 0.3429, one-way Mann–Whitney U test. C) Immunoblotting of pAKT (Ser473), AKT, DUSP6 and PTEN of tumour lysates generated from KPC *Kras*$^{G12D/+}$ and KPC *Kras*$^{G12D/fl}$ PDAC from mice at clinical endpoint. ß-ACTIN was used as loading control. Each lane represents an individual mouse of the indicated genotype. D) Boxplots representing GSVA enrichment of individual tumours derived from KPC *Kras*$^{G12D/+}$ and KPC *Kras*$^{G12D/fl}$ mice aged to clinical endpoint, with alignment to Bailey classification subtypes – ADEX, immunogenic, squamous and progenitor. Boxes depict interquartile range, central line indicates median and whiskers indicate minimum/maximum values (KPC *Kras*$^{G12D/+}$, n = 5; KPC *Kras*$^{G12D/fl}$, n = 6). E) Heatmap of gene programmes (GP) of Bailey classification of KPC *Kras*$^{G12D/+}$ and KPC *Kras*$^{G12D/fl}$ tumours (KPC *Kras*$^{G12D/+}$, n = 5; KPC *Kras*$^{G12D/fl}$, n = 6). GPs significantly enriched in KPC *Kras*$^{G12D/fl}$ tumours are highlighted in red. *P = 0.0411 (GP6, GP7), *P = 0.026 (GP8), one-way Mann–Whitney U test. F) Gene set enrichment analysis of tumour samples collected at clinical endpoint from KPC *Kras*$^{G12D/fl}$ and KPC *Kras*$^{G12D/+}$ tumours. Enrichment plots for immune-associated “Hallmark” signatures “Interferon Alpha Response”, “Interferon Gamma Response”, “Inflammatory Response” and “Allograft Rejection” are depicted.
