# Supplementary material for: KRAS Loss of Heterozygosity Promotes MAPK-Dependent Pancreatic Ductal Adenocarcinoma Initiation and Induces Therapeutic Sensitivity to MEK Inhibition
Source: Cancer Res. 2024 Oct 16;85(2):251–62. doi: 10.1158/0008-5472.CAN-23-2709 (PMC11733531; doi:10.1158/0008-5472.CAN-23-2709)
Supplement: Supplementary Figure 5 — Loss of wild-type KRAS sensitizes KPC KrasG12D/fl tumours to MEK1/2 inhibition. [file can-23-2709_supplementary_figure_5_suppsf5.pdf]

Suppl Figure 5

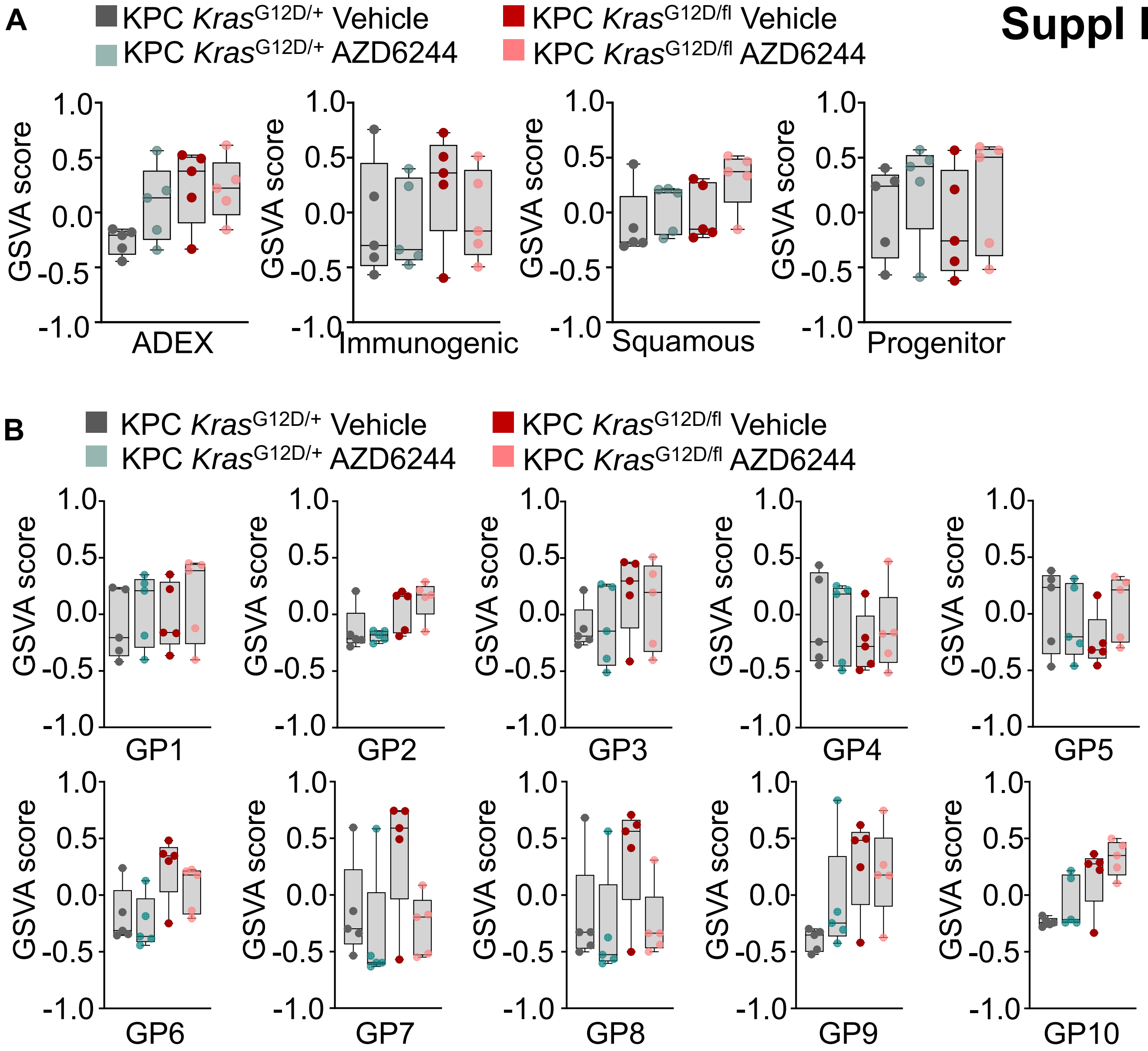

Supplementary Figure 5: Loss of wild-type KRAS sensitizes KPC *Kras*$^{G12D/fl}$ tumours to MEK1/2 inhibition. A) Boxplots representing GSVA enrichment of individual tumours derived from KPC *Kras*$^{G12D/+}$ and KPC *Kras*$^{G12D/fl}$ mice treated with Vehicle or AZD6244 as indicated from presence of palpable pancreatic tumour to clinical endpoint, with alignment to Bailey classification subtypes – ADEX, immunogenic, squamous and progenitor. Boxes depict interquartile range, central line indicates median and whiskers indicate minimum/maximum values (KPC *Kras*$^{G12D/+}$ vehicle, n= 5; KPC *Kras*$^{G12D/+}$ AZD6244, n = 5; KPC *Kras*$^{G12D/fl}$ vehicle, n= 5; KPC *Kras*$^{G12D/fl}$ AZD6244, n = 5). B) Boxplots representing enrichment of gene programmes (GP) from individual tumours derived from KPC *Kras*$^{G12D/+}$ and KPC *Kras*$^{G12D/fl}$ mice treated with Vehicle or AZD6244 as indicated from presence of palpable pancreatic tumour to clinical endpoint, with alignment to Bailey classification. Boxes depict interquartile range, central line indicates median and whiskers indicate minimum/maximum values (KPC *Kras*$^{G12D/+}$ vehicle, n= 5; KPC *Kras*$^{G12D/+}$ AZD6244, n = 5; KPC *Kras*$^{G12D/fl}$ vehicle, n= 5; KPC *Kras*$^{G12D/fl}$ AZD6244, n = 5).
