# Supplementary material for: KRAS Loss of Heterozygosity Promotes MAPK-Dependent Pancreatic Ductal Adenocarcinoma Initiation and Induces Therapeutic Sensitivity to MEK Inhibition
Source: Cancer Res. 2024 Oct 16;85(2):251–62. doi: 10.1158/0008-5472.CAN-23-2709 (PMC11733531; doi:10.1158/0008-5472.CAN-23-2709)
Supplement: Supplementary Figure 6 — MEK1/2 inhibition in KPC KrasG12D/fl mice with established tumours reverses enrichment of immune response related gene programmes. [file can-23-2709_supplementary_figure_6_suppsf6.pdf]

Suppl Figure 6

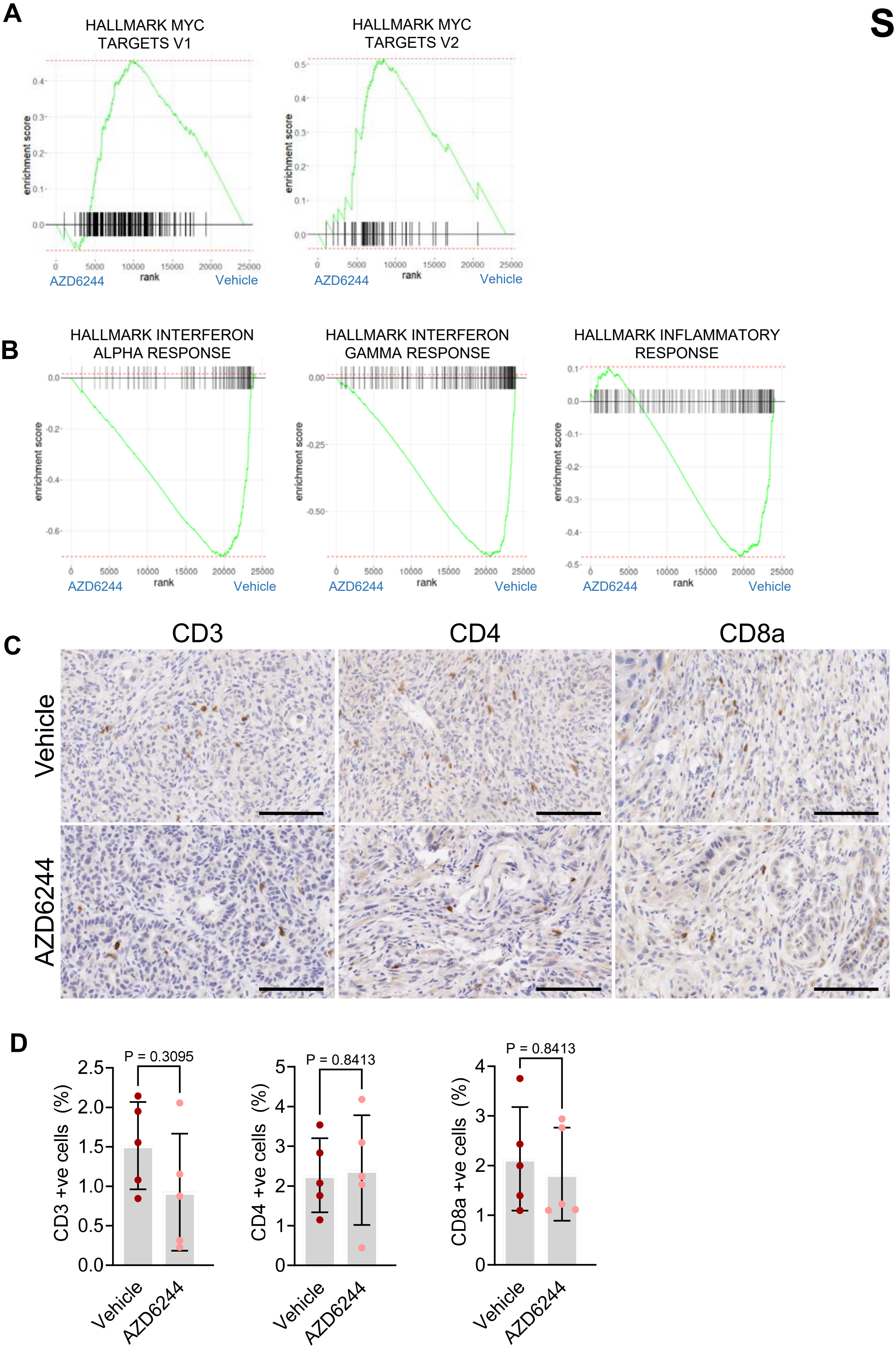

Supplementary Figure 6: MEK1/2 inhibition in KPC *Kras*$^{G12D/fl}$ mice with established tumours reverses enrichment of immune response related gene programmes. A) Gene set enrichment analysis of tumour samples collected at clinical endpoint from KPC *Kras*$^{G12D/fl}$ mice treated with vehicle or AZD6244 from presence of palpable pancreatic tumour. Enrichment plots for "Hallmark" signatures "Myc-targets V1" and "Myc-targets V2" are depicted. B) Gene set enrichment analysis of tumour samples collected at clinical endpoint from KPC *Kras*$^{G12D/fl}$ mice treated with vehicle or AZD6244 from presence of palpable pancreatic tumour. Enrichment plots for immune-associated "Hallmark" signatures "Interferon Alpha Response", "Interferon Gamma Response", "Inflammatory Response" and "Allograft Rejection" are depicted. C) Representative immunohistochemistry images of CD3, CD4 and CD8a from PDAC arising in KPC *Kras*$^{G12D/fl}$ mice treated with Vehicle or AZD6244 as indicated from presence of palpable pancreatic tumour to clinical endpoint. Representative of five mice per group. Scale bar 200 µm. D) Bar graphs showing quantification of proportion of CD3, CD4 and CD8a positive cells in PDAC tissue from KPC *Kras*$^{G12D/fl}$ mice treated with Vehicle or AZD6244 as indicated from presence of palpable pancreatic tumour to clinical endpoint (KPC *Kras*$^{G12D/fl}$ + Vehicle, n = 5; KPC *Kras*$^{G12D/fl}$ + AZD6244, n = 4). Data are mean ± s.e.m, Mann-Whitney U test, experiments represented in (C),
